# Reshaping the tumour immune microenvironment in solid tumours via tumour cell and immune cell DNA methylation: from mechanisms to therapeutics

**DOI:** 10.1038/s41416-023-02292-0

**Published:** 2023-04-28

**Authors:** Fengyun Zhong, Yilin Lin, Long Zhao, Changjiang Yang, Yingjiang Ye, Zhanlong Shen

**Affiliations:** 1 Department of Gastroenterological Surgery, Peking University People’s Hospital, 100044 Beijing, P. R. China; 2 Laboratory of Surgical Oncology, Beijing Key Laboratory of Colorectal Cancer Diagnosis and Treatment Research, Peking University People’s Hospital, 100044 Beijing, P. R. China

**Keywords:** Tumour immunology, Oncology, Epigenetics

## Abstract

In recent years, the tumour microenvironment (TME) of solid tumours has attracted more and more attention from researchers, especially those non-tumour components such as immune cells. Infiltration of various immune cells causes tumour immune microenvironment (TIME) heterogeneity, and results in different therapeutic effects. Accumulating evidence showed that DNA methylation plays a crucial role in remodelling TIME and is associated with the response towards immune checkpoint inhibitors (ICIs). During carcinogenesis, DNA methylation profoundly changes, specifically, there is a global loss of DNA methylation and increased DNA methylation at the promoters of suppressor genes. Immune cell differentiation is disturbed, and exclusion of immune cells from the TME occurs at least in part due to DNA methylation reprogramming. Therefore, pharmaceutical interventions targeting DNA methylation are promising. DNA methyltransferase inhibitors (DNMTis) enhance antitumor immunity by inducing transcription of transposable elements and consequent viral mimicry. DNMTis upregulate the expression of tumour antigens, mediate immune cells recruitment and reactivate exhausted immune cells. In preclinical studies, DNMTis have shown synergistic effect when combined with immunotherapies, suggesting new strategies to treat refractory solid tumours.

## Background

As mechanisms of cancer development have been gradually deciphered, tumour behaviours such as uncontrolled proliferation are considered to be the consequence of not only the accumulation of oncogenic alterations, but also epigenetic disruption. In fact, non-mutational epigenetic reprogramming has recently been included one of the fourteen hallmarks of cancer [1]. Epigenetic modifications inheritably control gene expression and are dynamically written and erased without changing the DNA sequence. Epigenetic control of tumour-related gene expression has been extensively studied in multiple tumour types [2–5], with some epigenetic factors accelerating cancer progression and some inhibiting it. In addition, plenty of diagnostic tools and therapeutic drugs related to epigenetic factors have been developed [6–8]. Among various epigenetic modifications, such as DNA methylation, histone methylation and acetylation, chromatin remodelling and noncoding RNA modification [9], DNA methylation is the most widespread and has been investigated the most. During carcinogenesis, global loss of DNA methylation occurs along with regional hypermethylation at CpG dinucleotide islands (CGIs) [10]. To date, drugs targeting aberrant DNA methylation, especially DNA methyltransferase inhibitors (DNMTis), have been employed to treat several haemopoietic malignancies such as myelodysplastic syndrome and acute myeloid leukaemia [11, 12]. However, they have not had curative effects in solid tumours.

In addition to the effects of tumour cells themselves, interactions between tumour cells and surrounding nontumor cells and tumour-infiltrating immune cells in the tumour microenvironment (TME) in particular, also play an indispensable role in cancer development. With advances in single-cell sequencing, an increasing number of immune cell subtypes in the TME have been identified, and researchers illustrated that immune cells can not only eradicate tumours but also be induced to take on a nonfunctional and even protumorigenic phenotype [13]. Infiltration of various immune cells causes tumour immune microenvironment (TIME) heterogeneity, and results in different therapeutic effects. As such, immunotherapies that are able to remodel TIME into one that is highly activated, including immune checkpoint inhibitors (ICIs), adoptive cell transfer and cancer vaccines, have been used to treat advanced or refractory cancers, such as non-small-cell lung cancer (NSCLC) [14], gastric cancer [15] and breast cancer [16], and have achieved longer survival. However, the majority of patients still fail to benefit [17, 18], and researchers attributed such failure to an inability of such treatments to reverse an “immune-cold” TIME.

Further studies have revealed that epigenetic factors participate in TIME regulation by affecting the expression of immune-related genes in both tumour cells and immune cells [19]. DNA methylation is not exceptional, and preclinical studies have revealed that DNMTis can remodel the TIME, elicit TIME reactivation and synergise with immunotherapies. In this review, we outline the mechanisms by which DNA methylation participates in carcinogenesis, and illustrate how DNA methylation and demethylation impact the differentiation of immune cells. Considering the very limited use of cellular immunotherapies and cancer vaccines in solid tumours, we mainly focus on ICIs, and we describe the mechanisms by which DNMTis reactivate antitumor immunity and their synergistic effects with ICIs. In addition, the specific DNA methylation landscape is related to the effect of immunotherapy. We describe potential DNA-methylation-based biomarkers and tools that can be used to evaluate the TIME status and predict the response to ICIs. We also summarise ongoing clinical trials of demethylating drug and immunotherapy combination treatment to highlight novel treatment strategies, and briefly discuss the limitations of current combination treatments.

## Overview of DNA methylation in cancer epigenome

### Mechanism of DNA methylation

**Fig. 1:**
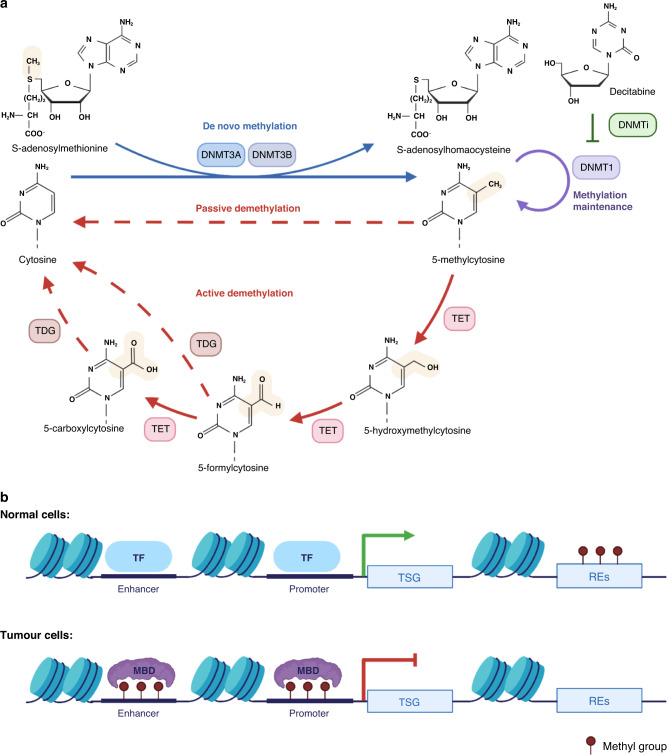
DNA methylation in the cancer epigenome. **a** DNA methylation cycle. DNA methylation mainly takes place at cytosines, and this process is dependent on S-adenosylmethionine and DNA methyltransferases (DNMTs) and can be suppressed by DNMT inhibitors (DNMTis). The reverse process, namely, DNA demethylation, can be both passively mediated by dilution of DNA replication and actively catalysed by ten-eleven translocation (TET) enzymes and thymine DNA glycosylase (TDG). **b** DNA methylation reprogramming during carcinogenesis. In normal cells, the enhancers and promoters of genes are often demethylated, while gene bodies, especially those of repetitive elements (REs) are hypermethylated; furthermore, tumour suppressor genes (TSGs) are normally transcribed. However, during carcinogenesis, global hypomethylation occurs concurrently with regional hypermethylation at enhancers and promoters, which is bound and interpreted by the methylation “reader” methyl-CpG binding domain (MBD) proteins, so that the expression of tumour suppressor genes is inhibited, and tumours can thus develop. TF transcriptional factor, MBD methyl-CpG binding domain, TSG tumour suppressor gene. Created with BioRender.com.

DNA methylation is a ubiquitous modification throughout the genome, found in areas including promoters, enhancers, gene bodies and intergenic regions [20]. A majority of methylation is found in CpG sequences in the genome, with the exception of CGIs and CGI shores that are located predominantly in promoters and enhancers [21]. Methylation in gene bodies often represents upregulated gene expression [22, 23], whereas abnormal methylation in CGIs, possibly due to DNA damage, often heralds transcriptional repression [22, 24]. For the modification process, methyl groups provided by S-adenosylmethionine (SAM) are added to target genes by “writers”, namely, DNA methyltransferases (DNMTs) [20, 25]. The reverse process, “demethylation”, can be either passive or active, with the latter involving the activity of ten-eleven translocation (TET) proteins and thymine DNA glycosylase (TDG) (Fig. 1a) [26].

### Profound changes of DNA methylation during carcinogenesis

In cancers, regional hypermethylation in promoters of tumour suppressor genes inhibits their expression and contributes to of cancer progression (Fig. 1b) [25]. Intriguingly, homeobox oncogenes have also been reported to be hypermethylated in cancer, leading to increased gene expression. Pan-cancer whole-genome bisulfite sequencing and locus-specific DNA methylation editing analyses have shown that such methylation is mainly established in DNA methylation canyon in gene bodies where there is a high density of CpGs [27]. In addition to regional hypermethylation, global loss of methylation is also common in cancer and it often occurs in repetitive elements in the genome, particularly in retrotransposons, such as long interspersed nuclear elements (LINEs) and short interspersed nuclear elements (SINEs) [25]. The function of this widespread hypomethylation in cancer progression is still unsettled, but it may result in chromosomal rearrangement and disrupted genome stability, since the density of unmethylated Alu elements has been found to be higher in cancer cells and related to low transcriptional activity and high chromosomal instability [28].

At the molecular level, such profound changes in DNA methylation are attributed to the disruption of the DNA methylation cycle. The activity of DNMTs is altered during carcinogenesis and drugs that inhibit the activity of DNMTs to rescue the aberrant methylation are somewhat effective in patients with some refractory diseases. The level of SAM also changes during carcinogenesis; therefore, targeting SAM and/or factors involved in its synthesis, such as methionine adenosyltransferases—which catalyse SAM production—is a prospective approach to treat cancer [29]. In addition, members of the TET family are putative cancer suppressors. For example, *TET2* loss-of-function mutation has been recognised as a vital cause of malignancies, especially leukaemia, because it reduces the level of 5hmC, resulting in an inability to maintain an unmethylated state of promoters and enhancers as 5hmC does in normal cells [21, 22]. Nevertheless, in solid tumours, functional mutations are rare, and TET expression varies among cancers and studies. Transcriptional and post-transcriptional regulation of TETs, as well as competitive substrates etc., more or less participate in the deregulation of TET proteins, and the detailed mechanisms have been reviewed elsewhere [30, 31]. Clarification of distinct roles of members of the TET family and their impact on solid tumours will provide strategies to manipulate these factors for cancer treatment.

Collectively, any alterations of factors involved in this cycle, including writers, readers and erasers, are likely to speed the progression of cancer; as such, targeting the key steps is a reasonable means to evaluate and treat patients, though further verification of the efficacy is needed.

## Immune cell differentiation is in company with methylation gain and loss

Hematopoietic stem cells are pluripotent and can differentiate into both myeloid and lymphoid lineages. During tumorigenesis, these immune cells acquire the expression of various markers, generating unique subtypes with diverse functions. It is now clear that dynamic changes in the global methylation level [32, 33], as well as methylation upregulation or removal at specific loci play a significant role in determining the fate of cells. For the purpose of regulating anti-tumour immune response, it is necessary to clarify the detailed mechanisms of DNA methylation in lymphocyte differentiation.

### The role of DNA methylation in T-lymphocytes differentiation

#### CD4^+^ T cells

For CD4^+^ T cell, following activation, demethylation occurs in almost all the differentiated subtypes, accompanied by an increase of 5hmC, suggesting the involvement of the TET family [34]. High-throughput DNA immunoprecipitation sequencing (DIP-seq) analysis indicates that 5hmC is mainly enriched at gene body and enhancer regions that putatively regulate genes involved in effector cell differentiation. With the assistance of lineage-specific TFs such as T-bet, the TET2 protein is recruited to 5hmC-containing regions and mediates DNA demethylation [34]. This results in the expression of specific cytokines or TFs and induces differentiation toward corresponding subtypes; for example, IL-4 leads to differentiation into Th2 cells, IL-17 leads to differentiation into Th17 cells, and FOXP3 leads to differentiation into tissue regulatory T (Treg) cells [34–36]. In the absence of TET2/3, FOXP3 expression is abrogated and Treg cell differentiation is impaired [37]. In addition to functional cells, to trigger robust and quick immune response, some naïve lymphocytes become inducible memory T cells. Deep epigenomes analysis has revealed that memory T-cell differentiation is defined by progressive global DNA demethylation of partially methylated domains (PMDs). PMDs are heterochromatic regions featuring repressive histone methylation marks, such as H3K27 trimethylation (H3K27me3) and H3K9 trimethylation (H3K9me3), and loss of DNA methylation correlates with increased proliferation. In addition, the FOXP1 gene is gradually methylated and FOXP1 expression is thus reduced during this process, so FOXP1 may be a naïve-keeping factor for CD4^+^ T cells [38]. Of note, after activation of T cells, regardless of the differentiation outcome, the overall level of DNMTs is diminished [34], whereas DNMT3A is a key factor sustaining the differentiated state and limiting the plasticity of CD4^+^ T cells; [38] these results indicate that DNMT3A has multiple functions in adaptive immunity.

#### CD8^+^ T cells

For CD8^+^ T cells, different stages of differentiation are also characterised by dynamic regulation of methylation, similar to the case for CD4^+^ T cells, and differential methylation in stage-specific regions leads to various outcomes. For instance, under the stress of strong and repetitive antigenic stimulation, effector T-cell-associated TFs tend to bind specific demethylated areas in genes such as *Ifng* and *Gzmb* to drive effector phenotype differentiation [20]. DNMT3A is considered crucial in CD8^+^ effector T-cell differentiation. It promotes methylation of the promoter of T-cell-specific transcription factor 7 (*Tcf7*) and silences its expression [39]. *Tcf7* encodes TCF1, a TF that is enriched in naïve T cells and central memory T cells but reduced in effector memory T cells and effector T cells, and silencing *Tcf7* hinders the renewal of stem-like T cell and the recall response of central memory CD8^+^ T cells [40]. Without DNMT3A, repression of naive-associated genes in effector CD8 T cells can be reversed, and early effector CD8^+^ T cells are biased to develop into long-lived memory precursor cells instead [39, 41]. Interestingly, TET2 was found to play a similar role to DNMT3A since its loss promoted memory cell differentiation [42]. In addition, in murine melanoma models, adoptively transferred TET2-deficient CD8^+^ T cells proliferated significantly, and more importantly, cytotoxicity was enhanced [43]. These findings imply that DNMTs and TETs can mediate T-cell dysfunction, and targeting them may promote differentiation toward functional antitumor T cells.

### DNA methylation affects myeloid cell differentiation

In order to trigger the tumoricidal effect of cytotoxic T lymphocytes (CTLs), the processing and presentation of tumour antigens by antigen-presenting cells (APCs) are indispensable; in this process, the major APCs are dendritic cells (DCs) and macrophages, both of which are derived from monocytes, and DNA methylation plays a role in their differentiation.

#### DCs

In normal monocyte-to-DC differentiation, TET2-mediated 5mC-to-5hmC conversion is also essential, as it is in T lymphocytes [44]. Stimulation of monocytes with granulocyte-macrophage colony-stimulating factor (GM-CSF) alone or together with interleukin-4 (IL-4) leads to macrophage or DC differentiation, respectively. IL-4 induces STAT6-mediated and TET2-dependent DNA demethylation of critical genes responsible for DC differentiation, such as *ITGB2* [45, 46]. Surprisingly, ex vivo experiment revealed an increase of DNMT1 and DNMT3A during monocyte-to-DC differentiation, while during DC maturation, downregulation of DNMT1 followed by DNMT3A upregulation was observed. This is likely attributed to the demethylation role of DNMTs by functioning as deaminases and base excision enzymes. DNMTi treatment did not affect the differentiation process but altered the surface marker and cytokine production of mature DCs [46]. Further research is required to illuminate the exact role of DNMTs in different stages of DC differentiation and maturation.

#### Macrophage

Genome-wide DNA methylation analysis of monocyte-to-macrophage differentiation revealed that not only loss but also gain of methylation occurred at very limited and localised regions especially at binding sites of TFs responsible for macrophage function [47]. An in vitro experiment revealed a phagocytic gene network containing over 100 discrete DNA regions that were demethylated and derepressed rapidly during monocyte-to-macrophage differentiation [48]. In terms of macrophages, polarisation towards either the activated M1 phenotype or the inhibitory M2 phenotype is also partly controlled by epigenetic regulation. Overexpression of DNMT3B induces macrophages polarisation towards M2 phenotype [26], while 5-Azacytidine (Aza) was found to induce macrophage to polarise toward the M1 phenotype and activate T cells in fat tissue to repress colorectal cancer (CRC) peritoneal carcinomatosis [49]. In addition, estradiol was identified to facilitate M2 macrophage polarisation during lung cancer progression by mediating DNMT1-induced p53 downregulation and consequent CCL5 upregulation as well as growth differentiation factor 15 downregulation [50].

#### MDSCs

Under the selection pressure of the TIME, myeloid-derived suppressor cells (MDSCs) stand out among a mass of immune cells, which are trained to be tolerogenic and protumorigenic and develop in a manner similar to that of DCs. MDSCs show unique DNA methylation patterns compared with DCs, and the methylation in MDSCs represses DC-related immunological gene signatures in a mechanism that is likely to be prostaglandin E2- and DNMT3A-dependent [51]. Analysis of tumour-infiltrating MDSC subsets revealed that the immature subtype was the major subtype with upregulated DNA methylation-mediated transcriptional silencing [52]. A study conducted by Daurkin et al. also showed that Aza administration was capable of reducing MDSCs and enhancing functional DCs, ultimately impeding tumour outgrowth in mice [53].

## DNMTis activate antitumor immunity by inducing viral mimicry in tumour cells

### Molecular mechanisms

**Fig. 2:**
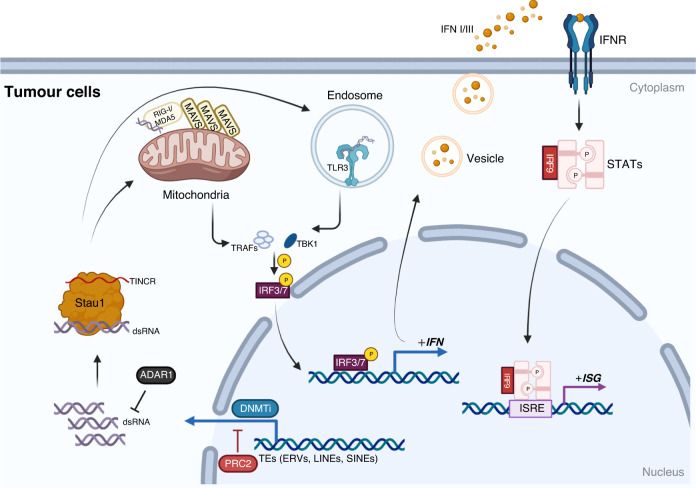
The viral-mimicry response induced by DNMTis. Upon DNMTi treatment, transposable elements (TEs) are induced to be transcribed and form double-strand RNA (dsRNA). The protein Stau1 and lncRNA TINCR are important for dsRNA stabilisation, and adenosine deaminase acting on RNA 1 (ADAR1) is able to degrade dsRNA. After being identified by toll-like receptor 3 (TLR3) in the endosome and retinoic acid-inducible gene protein I (RIG-I) or melanoma differentiation-associated protein 5 (MDA5) in the cytoplasm, dsRNA hierarchically activates the expression of type I/III interferons (IFN-I/III). Upon recognition of IFN-I/III, JAK-STAT pathway is activated, and Phosphorylated STAT1/2 bind IRF9 and translocate into the nucleus to bind IFN-stimulated response elements (ISRE) to activate interferon-stimulated gene (ISG) transcription. PRC2 polycomb repressive complex 2, MAVS mitochondrial antiviral signalling protein, TLR3 toll-like receptor 3, TRAFs TNF receptor-associated factors, TBK1 TANK binding kinase 1, IRF interferon regulatory factor. Created with BioRender.com.

In multiple studies of solid tumour models and clinical trials, exposure to demethylating agents such as the DNMTi Aza has been shown to increase the expression of MHC I and tumour antigens such as melanoma-associated antigen 1 and cancer-testis antigens (CTAs) [54–56], thereby enhancing antitumor immunity and even the efficacy of ICIs. The underlying mechanism is that demethylating drugs augment the transcription of transposable elements (TEs), including endogenous retroviruses (ERVs), LINEs and SINEs, especially inverted-repeat Alu elements, and induce double-stranded RNA (dsRNA) formation (Fig. 2) [57–59]. These TEs are generally distributed in intergenic regions and the evolutionarily youngest elements of the genome [60]. In normal development, TEs are often involved in building heterochromatin, and the human silencing hub (HUSH) complex cooperates with tripartite motif-containing protein 28 (TRIM28) to silence TE expression through epigenetic mechanisms [61].

Sensors of dsRNA, such as toll-like receptor 3 (TLR3) in the endosome and RNA helicases retinoic acid-inducible gene protein I (RIG-I) or melanoma differentiation-associated protein 5 (MDA5) in the cytoplasm recognise dsRNA and mediate hierarchical activation of downstream signals, ultimately triggering the type I/III interferon (IFN-I/III) response, which normally defends against viruses, in the absence of infection [57–60, 62, 63]. During this process, staufen1 protein and lncRNA TINCR are both essential to stabilise ERV RNA, low levels of which have been found to be correlated with inferior efficacy of DNMTis [64]. Similarly, the DNA signalling pathway can also be targeted during DNMTi administration, as the cyclic GMP–AMP synthase (cGAS)-stimulator of IFN genes protein (STING) pathway is sensitised after *STING* gets demethylated by zebularine [65]. In addition, genetically or pharmacologically induced demethylation is also able to cause hypoxia-inducible transcription factors (HIFs)-dependent expression of cryptic transcripts, which are more prone to trigger viral mimicry [66]. Generally, IFN-I/III is upregulated, and DNMTi is administered, they both can promote the demethylation and reactivation of silenced interferon-stimulated genes (ISGs) in cancer cells [67]. More importantly, secreted IFN-I/III is able to elicit the expression of ISGs in immune cells, promoting their infiltration and boosting their effector functions. The detailed regulation of immune cells by IFN-I/III has been summarised in multiple previous reviews [68, 69].

### Clinical evaluation and limitations

To substantiate these effects clinically, a gene set comprising 300 Aza-induced immune genes was used to classify multiple solid tumours into high, medium and low expression groups, with high and medium expression being associated with immune-reactive tumours and good prognosis [57]. Quantitative analysis of TEs also demonstrated a positive correlation between TE expression and immune cell infiltration, but TE overexpression was accompanied by PD-L1 expression and was indicative of poor prognosis in some patients [70]. However, from another perspective, patients with these features may be optimal candidates to benefit from ICIs, and in fact, the expression of some TEs has been considered as a possible predictor of good ICI response [71, 72].

Although promising, the rate of response to DNMTis is still limited. This is partly due to the expression of adenosine deaminase acting on RNA 1 (ADAR1) enzyme, which induces destabilization of dsRNA by A-to-I editing and reduces the viral-mimicry response. ADAR1 inhibition may enhance the effect of DNMTis and even overcome resistance to ICIs by inflaming the TME [63, 73]. Furthermore, chronically hypomethylated cancer cells appear to tolerate the viral-mimicry response. Tumour-intrinsic DNA demethylation represses immunomodulatory pathway gene expressions to drive immune evasion independently of mutation burden and aneuploidy, which may be the result of the formation of PMDs as opposed to highly methylated domains, which are enriched for immune-related genes with hypermethylation of their promoters [74].

Over and above these mechanisms of resistance, crosstalk with other epigenetic modifications should not be neglected. Polycomb repressive complex 2 (PRC2) is an essential substance mediating suppressive histone methylation and has been recently found to silence genes demethylated by DNMTis and mediate resistance to DNMTis in hepatocellular carcinoma [75]. In squamous cell carcinomas, recurrent loss-of-function mutations of the histone H3K36 methyltransferase NSD1 induces DNA hypomethylation but also tumour immune evasion. Despite increased levels of dsRNA, NSD1 loss drives inhibition of IFN-III response by deregulating histone methylation, which can be counteracted by inhibition of EZH2, the catalytic subunit of PRC2 [76]. In addition, protein arginine methyltransferase (PRMT) 7 inhibition in vivo results in reduced DNMT expression and increased RIG-I and MDA5 levels [77]. These results suggest crosstalk among various epigenetic modifications and the possible utility of combined treatment. In fact, PRC2-deficient cancers such as high-grade malignant peripheral nerve sheath tumour have been shown to benefit from DNMTi treatment the most because of their enhanced viral mimicry resulting from protein kinase R (PKR)-dependent dsRNA sensor activity [78], and combined treatment targeting both DNMTs and PRC2 was exactly indicated to augment antitumor immunity [75]. Further investigations are needed to delineate such interactions among various modifications clearly and harness them more efficiently to magnify the antitumor effect.

## DNA methylation reprogramming is vital in regulating immune cell trafficking and infiltration

**Fig. 3:**
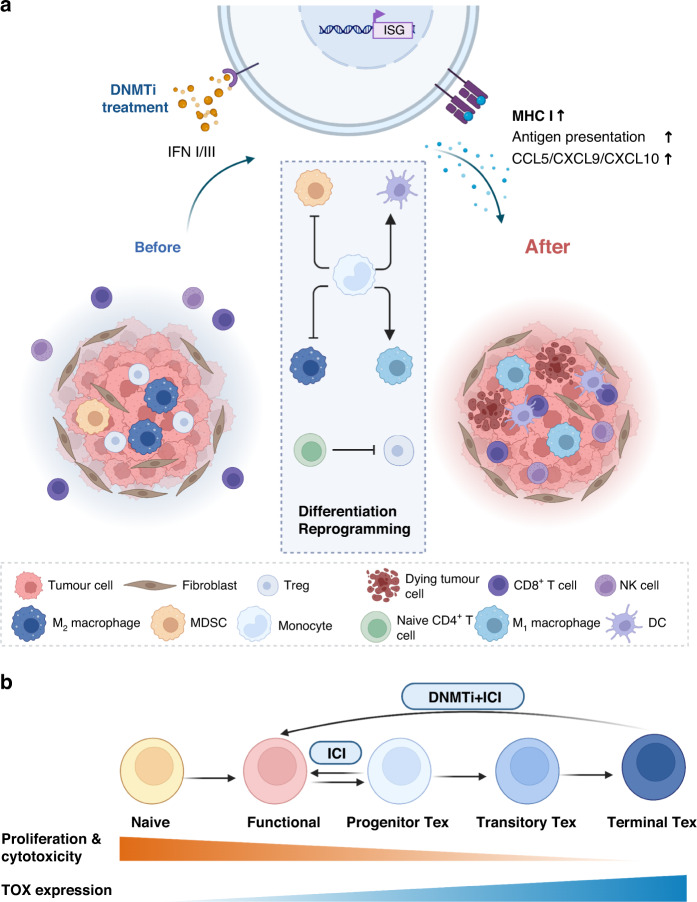
Remodelling of the TIME by DNMTi treatment. **a** Infiltration of antitumor immune cells after DNMTi treatment. In immune-cold tumours, functional antitumor immune cells are excluded from the tumour microenvironment (TME) and inhibitory immune cells such as myeloid-derived suppressor cells (MDSCs) are enriched. DNMTi treatment induces the expression of interferon-stimulated genes (ISGs), thereby increasing MHC-I expression, antigen presentation and chemokine levels. As a result, functional immune cells are able to infiltrate the TME and mediate tumoricidal reactions. In addition, DNMTi treatment also promotes the differentiation of functional immune cells and inhibits that of inhibitory cells. **b** Reinvigoration of Tex upon DNMTi treatment. After repetitive antigen stimulation, CD8^+^ T cells gradually become exhausted (Tex cells), with decreased proliferation ability and cytotoxicity but increased TOX expression. Due to de novo DNA methylation, only progenitor Tex cells respond to immune checkpoint inhibitor (ICI) therapy such as PD-1 blockade, but when DNMTi treatment is concomitantly administered, terminal Tex cells can be reinvigorated, thereby enhancing the efficacy of ICI therapy. Created with BioRender.com.

The antitumor immune response depends to a large extent on the quantity and type of immune cells in the TIME, based on which tumours can be divided into “hot” and “cold” tumours. Hot tumours are enriched in functional immune cells, especially Th cells and CTLs, and are often associated with better prognosis and superior ICI response. As tumours progress, they gradually develop a set of immune escape mechanisms that exclude tumour-killing cells from infiltration, and DNA methylation also plays a part in this process (Fig. 3a) [79].

To mediate migration into the TME, chemokines act as navigators to attract immune cells. Among chemokines, the chemokines CXC ligand (CXCL) 9 and CXCL10 have been consistently proven to be positively correlated with T-cell infiltration [79]. However, in murine ovarian cancer models, researchers have found that DNMT1-mediated DNA methylation inhibits the homing of Th1 cells by repressing the tumour production of CXCL9 and CXCL10, and thus disturb the trafficking of CD8^+^ effector T cells. Patient outcome was also found to be negatively related to DNMT1 expression [80]. In addition, chemokine CC ligand (CCL) 5 is also involved in the chemotaxis of T cells. Co-expression of CCL5 and CXCL9 predicts better survival and response to ICIs. Nonetheless, cancer cells negatively regulate *CCL5* expression to escape the surveillance of effector cells by DNA methylation [81]. Apart from T cells, the DNA methylation-mediated decrease in chemokine levels has an impact on other immune cells as well. For instance, macrophage infiltration in small-cell lung cancer (SCLC) is significantly decreased by epigenetic repression of CCL2 mediated by histone and DNA methylation [82]. A similar mechanism also exists for DCs and hampers the activation of the adaptive immune response.

In addition to impaired production of chemokines, the infiltration of immune cells can be negatively influenced by other mechanisms. Immune synapses are transient but necessary complexes that determine the activation of T cells. Immune synapse-related genes comprise immune checkpoint genes (ICGs) and costimulatory genes (CSGs). Disproportionate hypermethylation of CSGs and hypomethylation of ICGs are associated with impaired infiltration of T cells and worse survival [83]. Moreover, DNA methylation/demethylation-related activation of inhibitory immune cells such as MDSCs and Treg cells, as described above, will further promote the formation of an inhibitory TIME and impede the migration of cytotoxic immune cells [84].

Since DNA methylation has such a substantial effect on immune cell infiltration, DNMTis can naturally rescue the “cold” TIME. Preclinical models have demonstrated that CD8^+^ T cells and NK cells are more likely to infiltrate the TIME after administration of the DNMTi zebularine [65]. In another in vitro study, decitabine treatment enhanced the secretion of CXCL10 and CCL5 by clear cell renal cell carcinoma cells and increased T-cell infiltration [85]. DNMTi administration is also implicated in reinstating macrophage infiltration and M1 phenotype polarisation, ultimately suppressing tumour growth [82]. This in vitro evidence suggests that DNMTis employ mechanisms that can boost antitumor immunity by enhancing immune cell infiltration.

## DNA methylation and immune cell exhaustion

### De novo DNA methylation establishes the stable exhaustion state of immune cells

To evade immune system surveillance, in addition to inhibiting infiltration of immune cells, tumours also inhibit the activities of immunoreactive cells, leading to their exhaustion and death. For example, continuous stimulation of CD8^+^ effector T cells in chronic viral infection and cancer results in gradual loss of effector cytokines and abrogation of cytotoxic capability, along with decreased proliferative ability and increased expression of inhibitory receptors such as programmed cell death 1 (PD-1), a state generally known as T-cell exhaustion [56]. ICIs can reinvigorate exhausted T (T_ex_) cells, but the effect is not sustainable [86], and in one study, only ~10% T_ex_ cells fully responded to PD-1 blockade; [87] these results partly explain the failure of ICIs in patients with refractory cancer. Further studies have revealed that T_ex_ cells can be divided into progenitor T_ex_, transitory T_ex_ and terminal T_ex_ cells, and these cells exist on a trajectory; differentiation along the trajectory occurs in parallel with prolonged antigen exposure and a gradual increase in the expression of TOX, which is considered the key regulator of exhaustion. Progenitor T_ex_ cells are more proliferative and characterised by higher expression of *Tcf7* (like stem cells) than terminal T_ex_ cells, and more importantly, terminal T_ex_ cells are fixed in a dysfunctional state and unable to respond to ICI, whereas progenitor T_ex_ cells are reprogrammable(Fig. 3b) [87, 88].

In depth analysis of the establishment of exhaustion has indicated that multiple epigenetic mechanisms are involved, including histone methylation and acetylation, chromatin accessibility and DNA methylation. In terms of DNA methylation, Ghoneim et al. discovered that de novo DNA methylation written by DNMT3A was key in establishing a stable exhausted state and inhibited ICI-mediated T-cell rejuvenation; furthermore, many methylated regions were found to be correlated with TCR and IL-7/IL-2 signalling, especially regions in *Ifng* and *Myc*, as well as naïve-associated genes such as *Tcf7* [88, 89]. Intriguingly, Yang et al. characterised different subtypes of tumour-infiltrating lymphocytes (TILs) in CRC, and revealed that CD39^+^ CD103^+^ tumour-reactive TILs also expressed exhaustion markers such as PD-1 and Havcr2. DNA methylation profiling identified the demethylation status of their genes, and the TF-binding sites of exhaustion-associated TFs such as NR4A1 were also hypomethylated [90]. In addition, similar to the case for T cells, chronic stimulation of adaptive NK cells through NKG2C also results in dysfunction and exhaustion with upregulation of the checkpoint molecules lymphocyte activating 3 (LAG3) and PD-1 and genome-wide alterations in DNA methylation [91]. These results support the significance of DNA methylation in establishing immune cell exhaustion.

### DNMTis enhance anti-tumour immune response by reactivating exhausted immune cells

To reverse the dysfunctional state, researchers have tried to impair the de novo methylation process. DNMT3A knockout was able to restore the proliferation and recall potential of terminal T_ex_ cells, and decitabine-treated chimeric antigen receptor (CAR) T cells also showed enhanced and prolonged antitumor activity, implying that DNMTi treatment might sensitise refractory tumours to immunotherapy and improve the prognosis of advanced cancer [89, 92].

DNMTis such as decitabine can function in other ways to restore the activity of immune cells as well. As we have mentioned, DNA methylation in gene bodies is strongly correlated with high expression, and researchers have discovered that 3’-UTR DNA methylation is involved in the upregulation of immune-related genes, such as the immune checkpoint Havcr2. Decitabine inhibited this process and reinvigorated cytotoxic T cells [93]. Loo et al. showed that demethylating drugs promoted CD8^+^ T-cell activation and their cytolytic ability, and selected the subpopulation with high expression of granzyme B and perforin. Mechanistically, the cells overexpressed NFATc1 short isoforms associated with cytotoxicity and had increased chromatin accessibility at canonical recognition motifs of TFs involved in enhancing the transcriptional programme related to anti-tumour immunity [94]. A single-centre open-label clinical trial in refractory advanced solid tumours observed increased activity and abundance of IFNγ + T cells upon low-dose decitabine treatment, with subsequent polarisation of Th1 cells, increased cytotoxicity of effector T cells, and most importantly, better patient outcomes [95]. Moreover, decitabine was found to reverse resistance to NK cell effects caused by tumour cell downregulation of the expression of the NKG2D ligands ULBP1 and ULBP3 [96].

In summary, immune cell exhaustion is a mechanism by which tumour cells evade the immune system, and the primary driver is chronic antigenic stimulation. DNA methylation at least partly contributes to the maintenance of the exhausted state and irresponsiveness to ICIs, which can be reversed by DNMTi therapy. Moreover, the hypomethylation of checkpoint genes induced by DNMTis is likely to enhance ICI treatment by increasing target expressions, thereby supporting the combination of demethylating drugs and ICIs for the treatment of advanced solid tumours.

## Implications for clinical applications related to DNA methylation and the TIME

### Possible biomarkers and tools to evaluate the TIME on the basis of DNA methylation

Given the limited efficacy of ICIs in cancer patients, scientists have aimed to identify biomarkers and tools to evaluate the TIME and predict the efficacy of ICIs [97–99]. From the perspective of DNA methylation, mny biomarkers and tools have been exploited and have shown fair predictive ability (Table 1). The most direct and easiest approach is to assess the methylation status and expression of genes, for example, genes encoding checkpoint receptors and ligands [100–104]. However, some studies have shown the limited utility of such tools [105]. The inconsistent findings are likely a result of a mixture of factors, as reviewed elsewhere [106], and remains a challenge impeding clinical application. Other biomarkers include promoter methylation of FOXP1 [107], Cytohesin 1 Interacting Protein (CYTIP), TNF superfamily member 8 (TNFSF8) [105], TNF receptor superfamily member 9 (TNFRSF9) [108] and so on. It is worth noting that methylation of enhancers may be more closely related to immune cell infiltration and ICI response than methylation of promoters [109], but thus far, specific biomarkers have yet to be identified, and further exploration is needed.

**Table 1:** Biomarkers and tools to evaluate TIME on the basis of DNA methylation.

Biomarkers/tools	Functions	Cancer types	References
Methylation status of promoters			
Checkpoint receptors and ligands (PD-1, PD-L1, PD-L2, LAG3, CTLA-4, etc.)	Hypomethylation: unfavourable survival but high immune infiltration and good response towards ICI	Prostate cancer, ccRCC, melanoma, etc.	[100–104]
FOXP1	Unmethylation: improved PFS and OS after immunotherapy	Metastatic NSCLC	[107]
CYTIP	Hypomethylation: favourable anti-PD-1 response, PFS and OS	NSCLC	[105]
TNFSF8	Hypomethylation: favourable anti-PD-1 response, PFS and OS	NSCLC	[105]
TNFRSF9	Hypomethylation: higher immune infiltration and superior OS after ICI	Melanoma	[108]
Bioinformatic tools			
MethylCIBERSORT	Providing accurate estimates of cellular composition in TME	Pan-cancer	[110]
MeTIL	Measuring TIL distributions and predicting survival and chemotherapeutic effect	Breast cancer, melanoma, lung cancer	[111]
DIMEimmune	Estimating CD4^+^ and CD8^+^ T-cell abundance and TILs scores	CNS tumours	[112]
Signatures and scoring systems			
EPIMMUNE	Positive: improved PFS	Metastatic NSCLC	[107]
DMS	High DMS: immune activation, increased TMB and enhanced efficacy of ICI	Gastric cancer	[113]
LMC-based machine learning classifier	Predicting patients’ response towards ICI	Metastatic melanoma	[114]

*ICI* immune checkpoint inhibition, *ccRCC* clear cell renal cell carcinoma, *PFS* progression-free survival, *OS* overall survival, *NSCLC* non-small-cell lung cancer, *DMS* DNA methylation score, *LMC* latent methylation components.

Thanks to advances in high-throughput technology and bioinformatics analysis, researchers are now able to use large-scale DNA methylation data to infer the status of TIME. MethylCIBERSORT is a DNA methylation-based bioinformatic approach that provides accurate estimates of cellular composition in the TME and identifies tumours as immune hot or cold [110]. MeTIL can be used to assess the level of TILs and is superior to histopathological or immune marker assessment in predicting survival and chemotherapy efficacy in multiple cancers. Importantly, the evaluation can be completed by bisulfite pyrosequencing of small amounts of DNA from formalin-fixed, paraffin-embedded tumour tissue [111]. Differential Methylation Analysis for Immune Cell Estimation (DIMEimmune) is a similar but more reliable method since it sets gene expression data and immunohistochemistry-based lymphocyte counts as benchmarks [112].

Finally, DNA methylation-based signature and scoring systems, such as EPIMMUNE, an epigenomic profile based on a microarray DNA methylation signature in metastatic NSCLC, have also been employed to evaluate the response to ICI treatment [107]. Distinct immune subtypes of gastric cancer were identified based on a DNA methylation pattern and a scoring system called the DNA methylation score (DMS) was validated to be a promising biomarker of ICI efficacy [113]. In addition, a machine learning classifier based on CpG sites, specific for latent methylation components, was also predictive of response to ICI in metastatic melanoma [114].

In the future, these biomarkers and tools should be improved, enabling them to be applied across cancers and cancer stages, since many of them have only been tested and shown to be effective in specific stages and cancers. Moreover, with the help of single-cell technology, more markers of TILs and more specific subtypes related to immunotherapy should be identified to further improve the predictive ability.

### Demethylating drug and immunotherapy combination therapy is promising

In the previous sections, we discussed how DNA demethylation reshapes the TIME, including altering the differentiation direction of immune cells, promoting immune cells infiltration toward the TIME and reviving exhausted immune cells. Such remodelling is ubiquitously observed in various solid tumours; furthermore, DNA methylation is relatively controllable due to its reversibility and there is ample experience in the treatment of haematological malignancies. In addition, preclinically, in syngeneic BALB/c mouse models, high immune reactivity and low tumour growth were achieved by combining 5-aza-20-deoxycytidine (5-AZA-CdR) with the anti-cytotoxic T lymphocyte antigen 4 (CTLA-4) monoclonal antibody (mAb) 9H10 [115]. As a result, many efforts have been made to combine demethylating drugs with immunotherapy to produce synergistic effects, aiming to improve the prognosis of patients with advanced tumours. Table 2 summarises the registered clinical trials of combination therapies.

**Table 2:** Registered clinical trials combining demethylating drugs and immunotherapy in solid tumours.

ClinicalTrials.gov Identifier	Cancer types	Sample size	Demethylating drugs	Immunotherapy	Status	Phase
NCT05317000	Resectable HPV-associated HNSCC	50	5-azacytidine	Nivolumab	Not yet recruiting	Early P1
NCT02957968	Locally advanced HER2-negative breast cancer	47	Decitabine	Pembrolizumab	Active, not recruiting	P2
NCT05638984	Advanced ESCC	60	Decitabine	Tirelizumab	Not yet recruiting	P2
NCT02664181	Metastatic NSCLC	13	Tetrahydrouridine-decitabine	Nivolumab	Active, not recruiting	P2
NCT02961101	Relapsed or refractory malignancies (multiple)	250	Decitabine	Anti-PD-1 antibody	Unknown	P1/2
NCT03308396	Advanced kidney cancer	57	Guadecitabine	Durvalumab	Active, not recruiting	P1b/2
NCT05320640	Advanced solid tumours; relapsed/refractory non-hodgkin lymphoma	100	Decitabine	Anti-PD-1/PD-L1/CTLA-4 antibodies	Recruiting	P1/2
NCT03233724	Inoperable, or unresectable locally advanced or metastatic NSCLC and oesophageal carcinomas	85	Tetrahydrouridine-decitabine	Pembrolizumab	Recruiting	P1/2
NCT02959437	Advanced solid tumours	70	Azacitidine	Pembrolizumab; Epacadostat	Terminated	P1/2
NCT03179943	Recurrent/advanced urothelial carcinoma	21	Guadecitabine	Atezolizumab	Active, not recruiting	P2
NCT03445858	Relapsed or refractory solid tumours (excluding primary CNS tumours)	21	Decitabine	Pembrolizumab	Active, not recruiting	P1/2
NCT02608437	Metastatic melanoma	24	SGI-110 (guadecitabine)	Ipilimumab	Completed	P1
NCT02608268	Advanced/metastatic solid tumours	252	Decitabine	MBG453; PDR001	Terminated	P1/2
NCT05089370	Mucosal melanoma	30	Decitabine/Cedazuridine (DEC-C)	Nivolumab	Recruiting	P1/2
NCT01928576	NSCLC	101	Azacitidine	Nivolumab	Active, not recruiting	P2
NCT03206047	Recurrent ovarian, fallopian tube, or primary peritoneal cancer	75	SGI-110 (guadecitabine)	Atezolizumab; DEC-205/NY-ESO-1 fusion protein CDX-1401 vaccine	Active, not recruiting	P1/2
NCT02650986	Advanced malignancies expressing NY-ESO-1	15	Decitabine	TGFbDNRII-transduced autologous TILs	Active, not recruiting	P1/2
NCT05143125	Malignant tumours	60	Decitabine	NK cells	Recruiting	P1/2

*HNSCC* head and neck squamous cell carcinoma, *ESCC* oesophageal squamous cell carcinoma, *NSCLC* non-small-cell lung cancer, *CNS* central nervous system.

#### DNMTi plus anti-PD-1/PD-L1/CTLA-4 blockade

Ongoing and completed clinical trials have mainly been designed to include a DNMTi in combination with anti-PD-1/PD-L1/CTLA-4 blockade, and frequently used DNMTis include Aza, decitabine and the second-generation DNMTi guadecitabine. Despite all the advantages of combination treatment at the micro level, as discussed in previous sections, the outcomes of several clinical trials have been inconsistent. Generally, there has not been sufficient improvement in patient prognos. A study of pembrolizumab plus oral Aza CC-486 as treatment for advanced NSCLC patients found no improvement in PFS and OS [116]. A similar negative result was also observed in the Phase II METADUR trial in platinum-resistant ovarian cancer [117]. Even in the clinical trials that have had positive outcomes, patient response has only been mildly improved [118–120]. To explore the underlying reasons for such an unsatisfactory response, studies have assessed the methylation status and TIME after treatment. The methylation level of tumours was minimally altered in the METADUR trial [117], whereas in another trial, there was a 3% objective response rate (ORR) and global demethylation concomitant with increased gene expression, and there were increased levels of infiltrating TILs [120]. The reasons for such differences remain obscure, but the limited response despite increased TILs infiltration in the latter trial is indicative of additional mechanisms mediating resistance, such as Treg infiltration and IL-17 pathway activation. CyTOF analysis indicated that the patients with a durable clinical benefit or response (CBR) had more abundant naïve and central memory CD4^+^ T cells as well as classical monocytes in the TIME. Sufficient baseline levels of CD8^+^ T cells and CD20^+^ B cells and the presence of tertiary lymphoid structures were also associated with durable CBR, in line with trials using ICIs in melanoma and renal cell carcinoma [118, 121].

Safety and tolerability are also important. In the trial in refractory NSCLC we mentioned above, more adverse events were observed in the combination group compared with the group that received pembrolizumab treatment alone, and these adverse events gave rise to dose interruptions, possibly impacting the response rate [116]. However, in other clinical trials [118–120, 122], combination regimens were mostly safe and well tolerated. The differences in drug delivery methods, drug dosage and times of administration, as well as the general condition of the recruited patients may account for the differences.

#### Promising novel approaches that combine DNA methylation regulation and immunotherapy

**Fig. 4:**
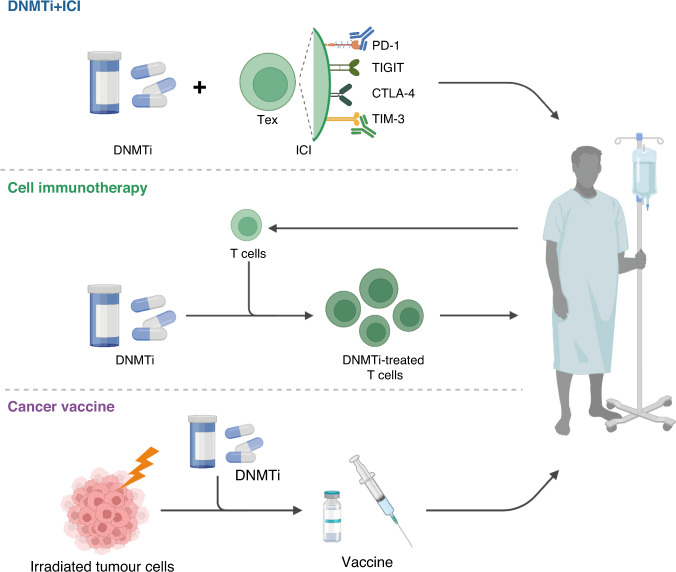
Combination therapy with DNA demethylating drugs and immunotherapy. Common immunotherapies include immune checkpoint inhibitor (ICI) therapy, cell immunotherapy and cancer vaccine; most of these therapies are still being tested in clinical trials, but a limited response has been observed in solid tumours. Some preclinical experiments have validated that DNMTi therapy can enhance immunotherapy in solid tumour treatment, and researchers have attempted to apply such combination therapy in patients. It is worth noting that for ICI, apart from common targets, including PD-1 and CTLA-4, other checkpoints such as TIM-3 and TIGIT are also promising targets. Created with BioRender.com.

In addition to these classical combination strategies, there are some novel targets and approaches (Fig. 4). For instance, checkpoint receptors include not only PD-1, PD-L1 and CTLA-4 but also TIM-3, LAG3, TIGIT and others, and their expression can be inhibited as well (NCT02608268). Moreover, adoptive cell transfer is another potential immunotherapy strategy (NCT02650986; NCT05143125), and has been tested in leukaemia. In a clinical trial in recurrent glioblastoma multiforme, adoptive DNMTi-treated CD4^+^ T cells expressed a large quantity of endogenous cancer–testis antigens, and were able to induce the generation of CTLs and NK cells, thereby inducing tumour regression in a few patients [123]. CAR T-cell therapy is the most common cellular immunotherapy, but DNMT3A-related exhaustion can occur and give rise to treatment failure. As shown in B-cell malignancies, DNA methylation status of patient CAR T cells determines the efficacy [124]. In preclinical studies in solid tumours, DNMT3A knockout or low-dose decitabine priming can retain the proliferative and cytolytic ability of CAR T cells, and establish effective recall responses [92, 125], so testing the effect on patients in the future is warranted. Cancer vaccines are another type of immunotherapy that is being increasingly studied. In a mouse model, hypomethylating agents were able to improve the antitumor effect of an irradiated whole-cell CRC vaccine (GVAX) by inducing CTA expression and T-cell response [126]. Some researchers are trying to harness a similar strategy in a combination regimen with a DNMTi (NCT03206047).

To sum up, present combination of ICI plus DNMTi does not live up to researchers’ expectation due to limited efficacy and unsatisfactory safety and tolerability. The underlying reason should be further studied as we have discussed and other approaches are worth exploration and clinical trials.

## Discussion

Epigenetic regulation and immunotherapy are both research areas that are receiving increasing attention. In this review, we focused on one of the most common epigenetic modifications, DNA methylation, and its impacts on TIME remodelling in solid tumours. According to current studies, DNA methylation plays a significant role in controlling the differentiation, infiltration and activation status of immune cells. Tumours modulate DNA methylation to evade the surveillance of the immune system. For this reason, DNMTis have been applied in treatment, and studies have shown that they increase antitumor immunity by promoting ERV activation and viral-mimicry response. DNMTi treatment results in immune cell recruitment toward the TIME and reinvigoration of cytotoxic immune cells as well. Because of the many positive effects of DNMTi, combination treatment with DNMTis and immunotherapies in solid tumours is warranted. Nonetheless, the outcomes of clinical trials are still not ideal, including low response rate and a lack of safety and tolerability data.

In the future, more targets and the functions of DNMTs and TETs in normal and cancer settings need to be identified. This will help us design epidrugs with enhanced targeting precision and efficacy and reduced side effects. Second, methylation levels vary both within and between lesions. With the assistance of single-cell and spatial omics analysis techniques, the methylation characteristics of different tumour lesions can be deciphered and targeted to maximise the effect. Third, apart from targeting writers of DNA methylation, readers and erasers can also be targeted. Moreover, as we have mentioned in this review, DNA methylation interacts with other kinds of epigenetic modifications. This suggests that the importance of each modification in different cancers and patients needs to be clarified, to select the most appropriate agents, and even to combine different agents together. Finally, it is necessary to determine the best dosage, delivery method and schedule in clinical settings.
