# Correction to: Reshaping the tumour immune microenvironment in solid tumours via tumour cell and immune cell DNA methylation: from mechanisms to therapeutics

**DOI:** 10.1038/s41416-023-02304-z

**Published:** 2023-06-01

**Authors:** Fengyun Zhong, Yilin Lin, Long Zhao, Changjiang Yang, Yingjiang Ye, Zhanlong Shen

**Affiliations:** 1 Department of Gastroenterological Surgery, Peking University People’s Hospital, 100044 Beijing, P. R. China; 2 Laboratory of Surgical Oncology, Beijing Key Laboratory of Colorectal Cancer Diagnosis and Treatment Research, Peking University People’s Hospital, 100044 Beijing, P. R. China

**Keywords:** Tumour immunology, Oncology, Epigenetics

Correction to: *British Journal of Cancer* 10.1038/s41416-023-02292-0, published online 28 April 2023

The article “Reshaping the tumour immune microenvironment in solid tumours via tumour cell and immune cell DNA methylation: from mechanisms to therapeutics”, written by Fengyun Zhong et al., was originally published electronically on the publisher’s internet portal on 28 April 2023 without open access. With the author’ decision to opt for Open Choice the copyright of the article changed on 23 May 2023 to © Author(s) 2023 and the article is forthwith distributed under a Creative Commons Attribution 4.0 International License, which permits use, sharing, adaptation, distribution and reproduction in any medium or format, as long as you give appropriate credit to the original author(s) and the source, provide a link to the Creative Commons licence, and indicate if changes were made. The images or other third party material in this article are included in the article’s Creative Commons licence, unless indicated otherwise in a credit line to the material. If material is not included in the article’s Creative Commons licence and your intended use is not permitted by statutory regulation or exceeds the permitted use, you will need to obtain permission directly from the copyright holder. To view a copy of this licence, visit http://creativecommons.org/licenses/by/4.0.

The original article has been corrected.

